# Editorial: Integrated multi-omic studies of metabolic syndrome, diabetes and insulin-related disorders: mechanisms, biomarkers, and therapeutic targets

**DOI:** 10.3389/fendo.2024.1537554

**Published:** 2025-01-10

**Authors:** Jairam K. P. Vanamala, Venketesh Sivaramakrishnan, Srinivas Mummidi

**Affiliations:** ^1^ Department of Food Science, Pennsylvania State University, University Park, PA, United States; ^2^ The Pennsylvania State Hershey Cancer Institute, Penn State Milton S. Hershey Medical Center, Hershey, PA, United States; ^3^ Disease Biology Lab, Department of Biosciences, Sri Satya Sai Institute of Higher Learning, Prasanthi Nilayam, Andhra Pradesh, India; ^4^ Department of Health and Behavioral Sciences, Texas A&M University-San Antonio, San Antonio, TX, United States

**Keywords:** metabolic syndrome, diabetes, insulin, biomarkers, multi-omics

## Introduction

Traditional reductionist approaches have been successfully applied to gain knowledge on monogenic disorders and diseases. However, such strategies are inadequate to probe and understand complex diseases like diabetes, metabolic syndrome (MS), and insulin-related disorders, where multiple genes and systems are perturbed. Understanding such intricate interrelationships and crosstalk demands holistic or systems-level integration, which can be achieved through single-omics/integrated multi-omics approaches.

This Research Topic explores how single-omics and integrated multi-omics analyses are transforming our understanding of the complex web of mechanisms, biomarkers, and therapeutic targets in metabolic syndrome, diabetes, and insulin-related disorders. Unlike the reductionist approaches, single-omics/multi-omics techniques offer a holistic view of complex diseases, highlighting their potential to advance personalized medicine significantly and targeted therapies, offering new hope in the fight against these diseases.

## The multi-omics approach: a holistic perspective

Multi-omics integrates data from various high-throughput platforms, including genomics (DNA sequences), epigenomics (gene expression regulation), transcriptomics (RNA transcripts), proteomics (proteins), microbiomics (microbial ecosystems), and metabolomics (metabolic byproducts). This comprehensive approach enhances our understanding of diseases and identifies actionable biomarkers and therapeutic pathways. Thus laying the foundation for precision medicine.

By integrating data across these diverse molecular layers, multi-omics provides an unprecedented ability to uncover the complex interactions and causal mechanisms that drive health and disease. This integrative perspective enables researchers to move beyond isolated insights, unraveling the interconnected networks underlying disease progression and therapeutic responses.

## Contributions of single-omics/multi-omics studies to disease understanding

### Mechanistic insights and biomarker discovery

Single-omics analysis and multi-omics data integration have played a pivotal role in unraveling the complex pathways associated with metabolic syndrome, diabetes, and related disorders. For example, transcriptomic analyses have uncovered the molecular mechanisms underlying diabetic retinopathy, enabling the identification of potential biomarkers for early detection and intervention. These discoveries advance scientific understanding and improve early disease detection and patient-specific management.

Global and targeted metabolomics studies have further revealed specific amino acid and acylcarnitine levels associated with metabolic syndrome, offering promising avenues for disease stratification and management strategies. Beyond uncovering mechanisms, these insights fuel the development of innovative therapies tailored to individual patient profiles, highlighting the transformative potential of single-omics and integrated multi-omics approaches in precision medicine.

### Therapeutic targets and interventions

In addition to providing insights into mechanisms and discovering biomarkers, multi-omics studies have identified novel therapeutic targets. An illustrative example of the potential application of multi-omics studies is ferroptosis, a newly discovered regulated cell death process characterized by the iron-dependent accumulation of lipid hydroperoxides to lethal levels. Multi-omics integrative studies have the potential to unveil innovative treatment strategies by identifying shared regulatory features between ferroptosis and the pathogenic pathways involved in obesity, type 2 diabetes, and atherosclerosis, which could serve as novel therapeutic targets.

## Case studies from the Research Topic

### Transcriptional signatures of diabetic retinopathy

Zhang et al.’s study on the transcriptomic signatures of diabetic retinopathy patients exemplifies the power of multi-omics analyses in elucidating disease mechanisms. By comparing the transcriptomes of patients with non-proliferative diabetic retinopathy to those without, the study identified immune system-related diagnostic genes, offering new insights into disease pathogenesis and potential therapeutic interventions.

### Metabolomics and metabolic syndrome

Taghizadeh et al.’s study illustrates the power of metabolomics in elucidating the metabolic alterations associated with metabolic syndrome. They identified specific metabolic profiles in a large cross-sectional study demonstrating the potential of metabolomics in disease prediction and prevention strategies.

### Ferroptosis and metabolic diseases

Zhou et al.’s review on the role of ferroptosis in metabolic diseases provides a comprehensive overview of how targeting this cell death process could offer novel therapeutic approaches for managing obesity, type 2 diabetes, and atherosclerosis. The study highlights the intersection of ferroptosis with key pathogenic pathways, emphasizing its relevance as a therapeutic target.

### Pathogenic mechanisms of diabetic peripheral neuropathy

Zhu et al.’s exhaustive review of diabetic peripheral neuropathy (DPN) presents an integrative view of the disease’s pathogenesis. The discussion of various pathways involved in DPN and their therapeutic implications underscores the complexity of the disease and the necessity of multi-omics approaches in devising effective prevention and treatment strategies.

### Novel indices for assessing insulin resistance

The study by Shao et al. on the relationship between a novel index and insulin resistance attests to the potential of integrating omics data with clinical indices to enhance disease assessment and management. Exploring non-linear relationships and validating novel metrics against established methods highlights the ongoing evolution of disease diagnostics.

## Conclusion

Single-omics and integrated multi-omics studies have emerged as cornerstones in understanding and managing metabolic syndrome, diabetes, and insulin-related disorders. By providing holistic insights into disease mechanisms, facilitating biomarker discovery, and identifying novel therapeutic targets, these approaches are not only driving a paradigm shift but also instilling a sense of hope and optimism in how we approach complex diseases.

As we integrate multi-omics approaches with advances in computational biology and artificial intelligence, we stand at the cusp of a new era in disease management. This integrative strategy promises to revolutionize patient outcomes and our broader understanding of the underlying molecular mechanisms in health and disease.

By unraveling the intricate networks that govern disease progression, multi-omics approaches empower researchers and clinicians to move beyond reactive treatments and toward proactive, precision-based care. The continued development and application of these tools and transdisciplinary collaborations will redefine how we diagnose, prevent, and treat diseases. Ultimately, these innovations have the potential to improve lives by contributing to a deeper, more comprehensive understanding of human and animal health.

